# A phase 2a clinical study on the safety and efficacy of individualized dosed mebendazole in patients with advanced gastrointestinal cancer

**DOI:** 10.1038/s41598-021-88433-y

**Published:** 2021-04-26

**Authors:** S. Mansoori, M. Fryknäs, C. Alvfors, A. Loskog, R. Larsson, P. Nygren

**Affiliations:** 1 Department of Medical Sciences, Uppsala University, Uppsala, Sweden; 2 Uppsala Clinical Research Centre, Uppsala University, Uppsala, Sweden; 3 Department of Immunology, Genetics and Pathology, Uppsala University, Uppsala, Sweden; 4 Repos Pharma AB, Uppsala, Sweden

**Keywords:** Drug development, Targeted therapies, Gastrointestinal cancer

## Abstract

Mebendazole is used extensively for treatment of local gut helminthic and invasive echinococcus infections. Anticancer effects of mebendazole have been shown in experimental cancer models and in case studies in patients with advanced cancer. Given these observations, the aims of this study were to investigate safety and efficacy of individualized dosed mebendazole in the cancer indication. Patients with treatment refractory gastrointestinal cancer were treated with individualized dose adjusted mebendazole up to 4 g/day to target a serum concentration of 300 ng/ml. Efficacy and safety were assessed by CT-scans, clinical surveillance and blood sampling. Eleven patients were included in the study and 10 started the treatment phase. Two patients stopped treatment prior to and the remaining eight after tumour evaluation by CT-scan at 8 weeks, all due to progressive disease. Four patients also fulfilled criteria suggested for hyperprogression. Only five patients reached the target serum-mebendazole concentration. No severe adverse effects were observed. Individualized dose adjusted mebendazole is safe and well tolerated in patients with advanced cancer but all patients experienced rapid progressive disease. New approaches such as prodrug development and combination with other anticancer drugs seem needed for further exploration of mebendazole as an anticancer drug.

## Introduction

Mebendazole (Mbz) has been used extensively during long time for local gut helminthic infections at low dose (Vermox; 100 mg × 2 for up to 5 days) but also at considerably higher doses, e.g. 40 mg/kg per day, for months to years against invasive echinococcus infections^1–3^. However, recent research has indicated that Mbz also produce anticancer effects. Thus, Mbz has anti-tumour activity against various tumour cell lines in vitro at concentrations achievable in patients on high dose treatment^4,5^. In xenograft models in rodents of medulloblastoma, glioblastoma and melanoma, Mbz stops or retards tumour growth at high doses, i.e. 50 mg/kg/day by oral gavage^6,7^. Case studies in patients with metastatic adrenocortical carcinoma and colon cancer, respectively, indicate that the preclinical effect can be translated to the clinical situation without major safety concerns^8,9^.

The mechanism of action behind the antiparasitic effect of Mbz is believed to be mediated by microtubule disruption. Additional mechanisms of action suggested for the anticancer effect are caspase activation, angiogenesis inhibition, hedgehog inhibition, and inhibition of signal transducing kinases^10–12^. We recently found Mbz to also be an immunomodulating agent, e.g. acting to switch macrophages from tumour promoting M2 to tumour inhibitory M1 phenotype and to stimulate activated lymphocytes to target tumour cells, seemingly by activation of ERK^13–15^.

Since Mbz has been used in the clinic for many years for the treatment of helminthic infections there is a considerable amount of data published, also for its use at high dose for invasive infections. Long-term high-dose (≥ 40 mg/kg per day) treatment with Mbz for invasive parasitic invasion is generally well tolerated with transient hair loss, gastrointestinal problems, elevated but reversible transaminases and mild leukopenia and thrombocytopenia as the most common adverse events (AEs)^16^.

Thus, there was a strong rational for evaluating safety and efficacy of Mbz in this phase 2a clinical trial in cancer patients with advanced cancer no longer amenable for standard drug treatment. Given the well-known poor bioavailability of Mbz and the indications from our preclinical investigations and our case study that a Mbz concentration of close to 1 µM might be required for its anti-cancer activity, we applied individualized dosing based on intensive therapeutic drug monitoring (TDM) with the aim to reach this concentration in all patients.

## Patients and methods

### Overall study outlines and eligibility criteria

This study (14/08/2018, Clinicaltrial.gov, NCT03628079) was a prospective, single armed, open label phase 2a study in two parts: the pharmacokinetic (PK) phase (week-1) and the treatment phase (up to 16 weeks of continuous treatment). The study was conducted at one study site in Sweden (Department of Oncology, Uppsala University Hospital, Uppsala) in accordance with the Declaration of Helsinki. The ethical approval was reviewed by Swedish Ethics Review Board (Dnr 2017/470 and Dnr 2019/03309). Written informed consent for participation in the study as well as written informed consent for publication and was provided by all the participants before study enrolment.

Primary endpoints were safety profile of Mbz and anti-tumour efficacy measured as tumour response rate according to RECIST 1.1 and prolongation of time to tumour progression (TTP) compared with the treatment preceding Mbz. Mbz PK including number of patients reaching a steady-state serum-Mbz concentration in the range 250–350 ng/mL (i.e. close to 1 µM) were key secondary endpoints.

Given the early and explorative phase of development of Mbz as an anticancer drug, 30 evaluable patients were considered suitable for preliminary assessment of Mbz tolerance, anti-tumour efficacy and PK. Key inclusion criteria were at least 18 years of age, histologically confirmed diagnosis of squamous cell cancer or adenocarcinoma, including primary cancer of the liver, of the gastrointestinal tract or cancer of unknown origin, measurable disease according to RECIST 1.1, defined TTP on the standard/experimental treatment preceding the study treatment and locally advanced or metastatic disease not amenable to standard treatment, i.e. progress on standard therapy or observed/expected intolerance to standard therapy. Key exclusion criteria were anti-tumour therapy within 3 weeks prior to study drug administration day 1, ongoing infection or other major recent or ongoing disease, WHO performance status ≥ 2, Child–Pugh B or C liver function status if hepatocellular carcinoma and blood laboratory parameters indicating major organ dysfunction.

### Study performance

**Figure 1:**
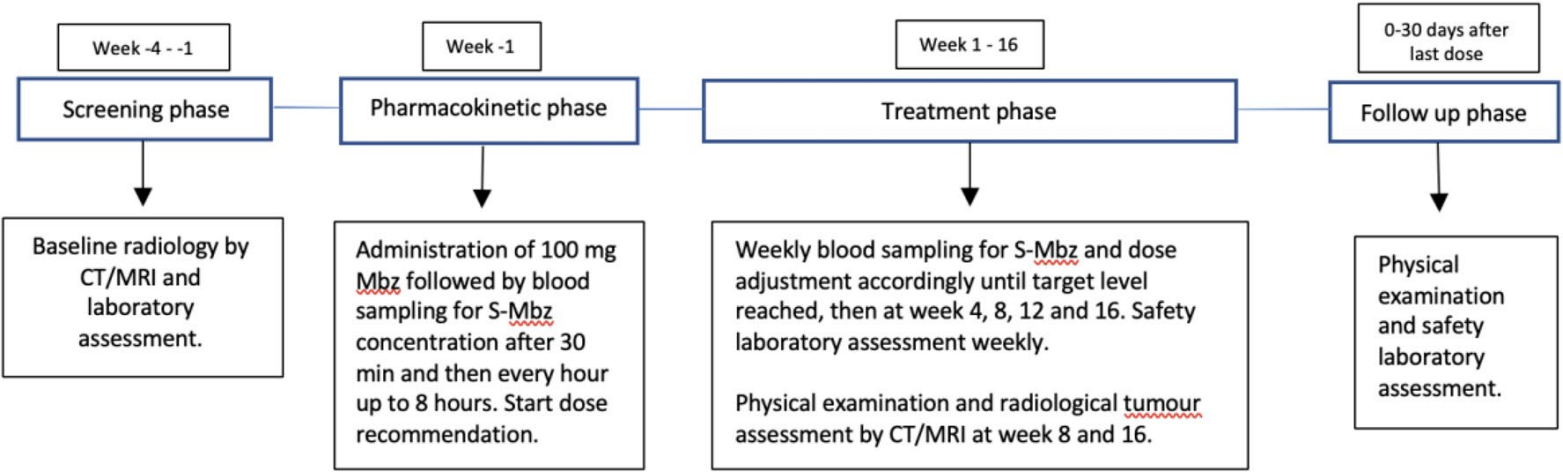
Flow chart of the study procedures. *CT* computerized tomography, *Mbz* mebendazole, *MRI* magnetic resonance imaging, *S* serum.

The patient related study procedures are illustrated in Fig. 1. TDM was used to adjust the Mbz dose of study drug in each patient. Based on prior experience (unpublished) in a pilot patient, the target therapeutic serum concentration of Mbz was estimated to be 300 ng/mL (1 μM; accepted range 250–350 ng/mL). Mbz was prepared specifically for the trial (Recipharm AB, Solna, Sweden) as powder in gelatin capsules with strengths 50, 100 and 200 mg/capsule to allow for individual dose adjustments. Mbz was always to be taken together with 40 mL Calogen Extra Shot (Nutricia AB, Stockholm, Sweden) nutritional soft-drink providing 16 g fat to allow for optimal drug uptake^17^.

In the PK phase week-1, following baseline assessments, i.e. tumour imaging by CT/MRI of abdomen and chest within 3 weeks, blood chemistry/haematology and baseline samples for translational research within 1 week, the patient took 1 capsule Mbz 100 mg, preferably within 1 h after breakfast, together with the nutritional soft-drink, at approximately 8 a.m. Monday. At pre-dose, 0.5 h, 1 h, 2 h, 3 h, 4 h, 6 h and 8 h after dose, blood samples were taken for analysis of serum-Mbz. If C_max_ exceeded a serum concentration of 400 ng/mL, the patient went off study since a too high drug exposure would be expected even from the lowest dose of Mbz available.

The treatment phase then started on Friday morning week-1 (study day 5) with a Mbz dose aimed to reach a C_max_ close to 300 ng/mL (1 μM; accepted range 250–350 ng/mL). The starting dose of Mbz was dependent on the C_max_ observed during the PK study phase and chosen based on a study specific dosing algorithm (Supplementary Table 1). Drug intake was to be as close as possible to 8 a.m. and 8 p.m. continuously. Serum-Mbz was measured again Monday week 1 (study day 8). Sampling for serum-Mbz concentration during the therapeutic study phase was adjusted and reduced based on the T_max_ observed in the PK-phase and the experience that T_max_ is shorter during repeated compared with single Mbz dosing^16^.

**Table 1:** Characteristics of the patients included.

Patient no	Age, sex	Diagnosis	Year of diagnosis	Locally advanced/metastatic disease	No of prior chemotherapy lines	Prior TTP (days)
101	56, female	Anal squamous cell carcinoma	2016	Yes/yes	2	73
102	60, female	Rectal adenocarcinoma	2016	Yes/yes	3	180
103	52, male	Rectal adenocarcinoma	2017	Yes/yes	2	99
104	62, male	Adenocarcinoma of colon	2017	No/yes	3	64
105	57, female	Adenocarcinoma of colon	2012	No/yes	5	220
106	48, female	Rectal adenocarcinoma	2016	No/yes	3	154
107	73, female	Adenocarcinoma of colon	2007	No/yes	6	117
108	69, male	Rectal adenocarcinoma	2009	Yes/yes	6	63
109	22, male	Hepatocellular carcinoma	2017	No/yes	2	300
110	74, male	Pancreatic carcinoma	2016	Yes/yes	4	56
111	43, male	Oesophageal squamous cell carcinoma	2016	No/yes	4	70

*TTP* time to tumour progression on the chemotherapy regimen preceding enrolment in this study.

The time-points for serum concentration sampling for assessment of C_max_ and dose adjustments were subsequently individualized based on the repeat-dose observations. Based on the PK result the dose was adjusted with start the following Friday at approximately 8 a.m. Dose changes were based on a study specific dosing algorithm (Supplementary Table 2). This procedure was repeated weekly until the target serum-Mbz concentration had been reached. The serum-Mbz level was then to be checked on Monday treatment week 4, 8, 12 and 16 as applicable, and if necessary adjusted as described above. The daily dose of Mbz was capped at 4 g.

Blood samples for the safety assessments were collected weekly during treatment. Blood sampling for cytokines and immune cells was done at baseline and then weekly until the target serum-Mbz concentration had been reached, and then at week 4, 8, 12 and 16.

Each patient was planned to receive 16 weeks of continuous Mbz but to stop earlier in the case of unequivocal signs of tumour progression. Tumour status according to RECIST was to be assessed radiologically by computerized tomography (CT), after 8 and 16 weeks on treatment unless there were unequivocal signs of tumour progression with clinical deterioration before any of these time points. Patients withdrawn from treatment due to progressive disease (PD) at 8 weeks but still included in the trial would undergo radiological tumour evaluations at the discretion of the investigator.

Tumour growth kinetics (TGK) was calculated from repeated CT-scans as described by Saâda-Bouzid^18^.

Thus, TGK was calculated as: *TGK* = (*Sum of longest diameters of target lesions (SLD) at treatment evaluation* − *SLD prior to start of treatment*)/(*treatment duration*).

TKG_ratio_ was calculated as: *TKG while on Mbz treatment/TKG just prior to start of Mbz.*

Hyperprogressive disease was defined as a TKG_ratio_ ≥ 2 as suggested by Saâda-Bouzid^18^.

Treatment was temporarily stopped for up to 3 weeks for adverse events to recover (max 2 temporary stops were allowed). Treatment was permanently stopped upon unacceptable adverse event, clinical deterioration or patient withdrawal of consent.

## Results

### Patients

The study was planned to include 30 evaluable patients. Since the patients to be included were expected to be heavily pre-treated an objective radiological tumour response rate of ≥ 10% was considered promising for Repos MBZ as an anticancer drug. Inclusion of 30 patients would result in a 95% confidence interval around this response rate of 2–26% which was arbitrarily considered to allow for conclusions on drug efficacy in a phase 2a trial. However, after enrolment of 11 patients, the study was prematurely discontinued. The reasons for stopping the study were difficulties to reach and maintain the target serum-Mbz concentrations despite TDM based dosing up to the daily maximum of 4 g and absence of treatment benefit. Five females and 6 males were enrolled in the study. The mean age of the patients was 55.8 years ranging from 22 to 73 years. The mean weight was 71.7 kg (range 47–99 kg) and the mean BMI was 23.8 kg/m^2^ (range 17–33 kg/m^2^). Seven patients were diagnosed with colorectal adenocarcinoma, 1 with anal squamous cell carcinoma, 1 with hepatocellular carcinoma, 1 with pancreatic carcinoma and 1 with oesophageal squamous cell carcinoma. Five patients had locally advanced disease and all patients had metastatic disease. The year of diagnosis ranged from 2007 to 2017, and the majority of patients were diagnosed in 2016. At screening, 4 patients had WHO performance status 0 and 7 patients had WHO 1. Patient demography is summarized in Table 1.

Seven of the patients had cancer surgery performed in the past, most often with a curative intent. All patients had received prior chemotherapy (mostly irinotecan, oxaliplatin, capecitabine, folinic acid and fluorouracil) before enrollment in the study. The number of lines of prior chemotherapy ranged from 2 to 9. The median TTP on the chemotherapy regimen preceding enrolment in the current study was 99 days (range 56–300).

For the 10 patients reaching the treatment phase the median time on treatment was 52 days (range 14–91). Treatment compliance estimated from patients’ diary drug amount returned ranged 82–100%. All patients starting treatment phase were withdrawn from study treatment due to radiological PD and/or clinical deterioration. Patient 111 had a stroke on the day after the first test dose of Mbz was administered during study week-1 and never started the treatment phase.

### Efficacy

#### Tumour response

In total 8 patients were evaluated with CT for tumour response at week 8 and the overall tumour response assessment showed PD for 7 of these patients with new lesions in liver and/or lung. The CT at 8 weeks for patient 106 showed PD but with necrosis in some metastases, suggesting tumour response, why the treatment with Mbz was continued. A new evaluation of the tumour status was done at 16 weeks and the assessment showed PD and the treatment was discontinued at that point. Four patients (102, 103, 105 and 107) performed additional CT scans after discontinuation of treatment with Mbz at week 8. Three of these, all without treatment, showed further PD while patient 107 had restarted chemotherapy and showed tumour regression. Three patients (101, 104 and 111) terminated the study before week 8 and no radiological tumour evaluation after treatment start was performed. Two of these patients experienced clinical deterioration due to PD and patient 111 terminated the study already in the PK-phase due to stroke.

#### Hyperprogressive disease

**Figure 2:**
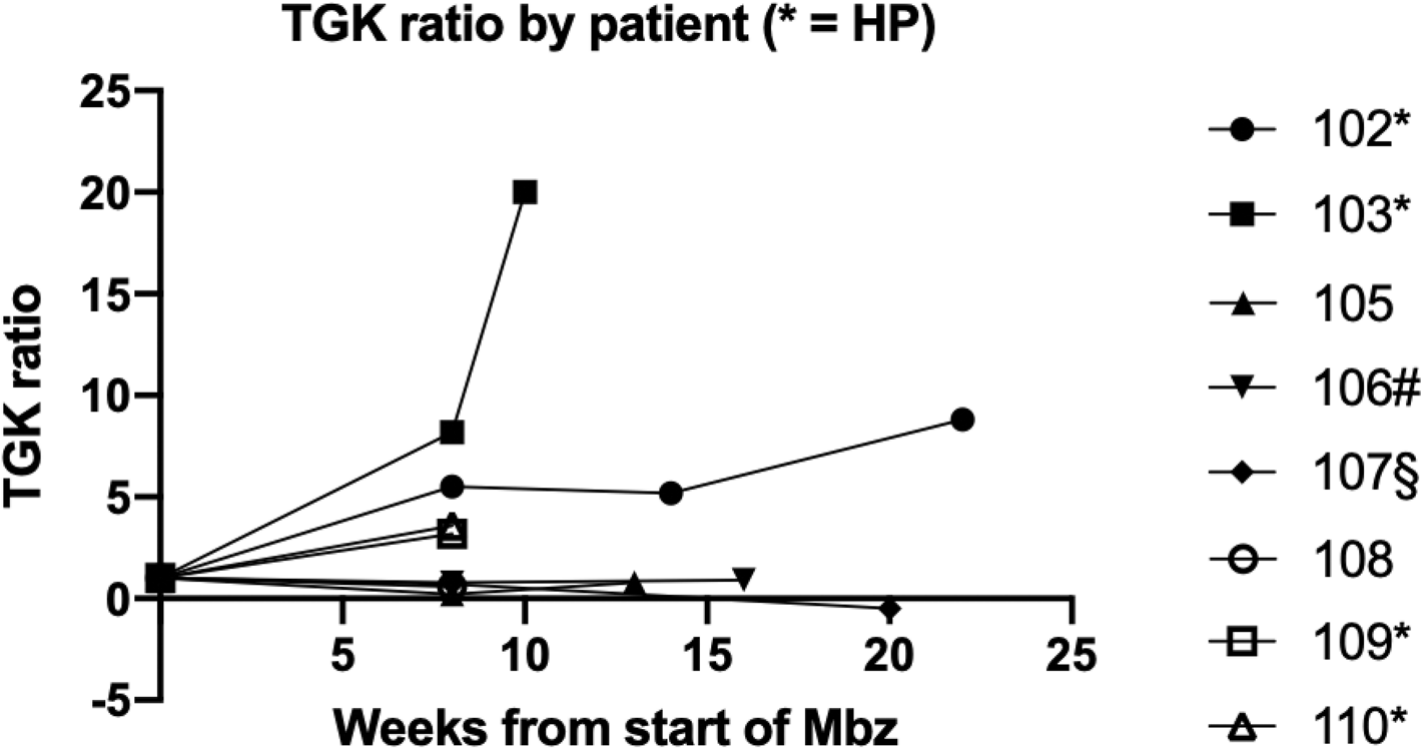
Graphical presentation of tumour growth kinetics (TGK) ratios during and after (as applicable) treatment with mebendazole (Mbz) for the radiologically evaluable patients. The TGK ratio pre-baseline was set to 1. TGK ratios were calculated by dividing the TGK from start of Mbz to w 8 on Mbz treatment, from w 8 to next radiological assessments and from this to the last assessment (patient 102), respectively, with the TGK pre-baseline. Pre-baseline TGK was calculated from CT scans while pts 103 and 106 were on chemotherapy whereas the remaining pts had no treatment. At post Mbz assessment patient 107 had restarted chemotherapy showed tumour regression as indicated by the negative TGK ratio. *Indicates patients fulfilling criteria for hyperprogression (HP) while on Mbz. ^#^This patient continued Mbz from week 8 to next radiological assessment. ^§^This patient had restarted chemotherapy from week 8 to next radiological assessment.

**Figure 3:**
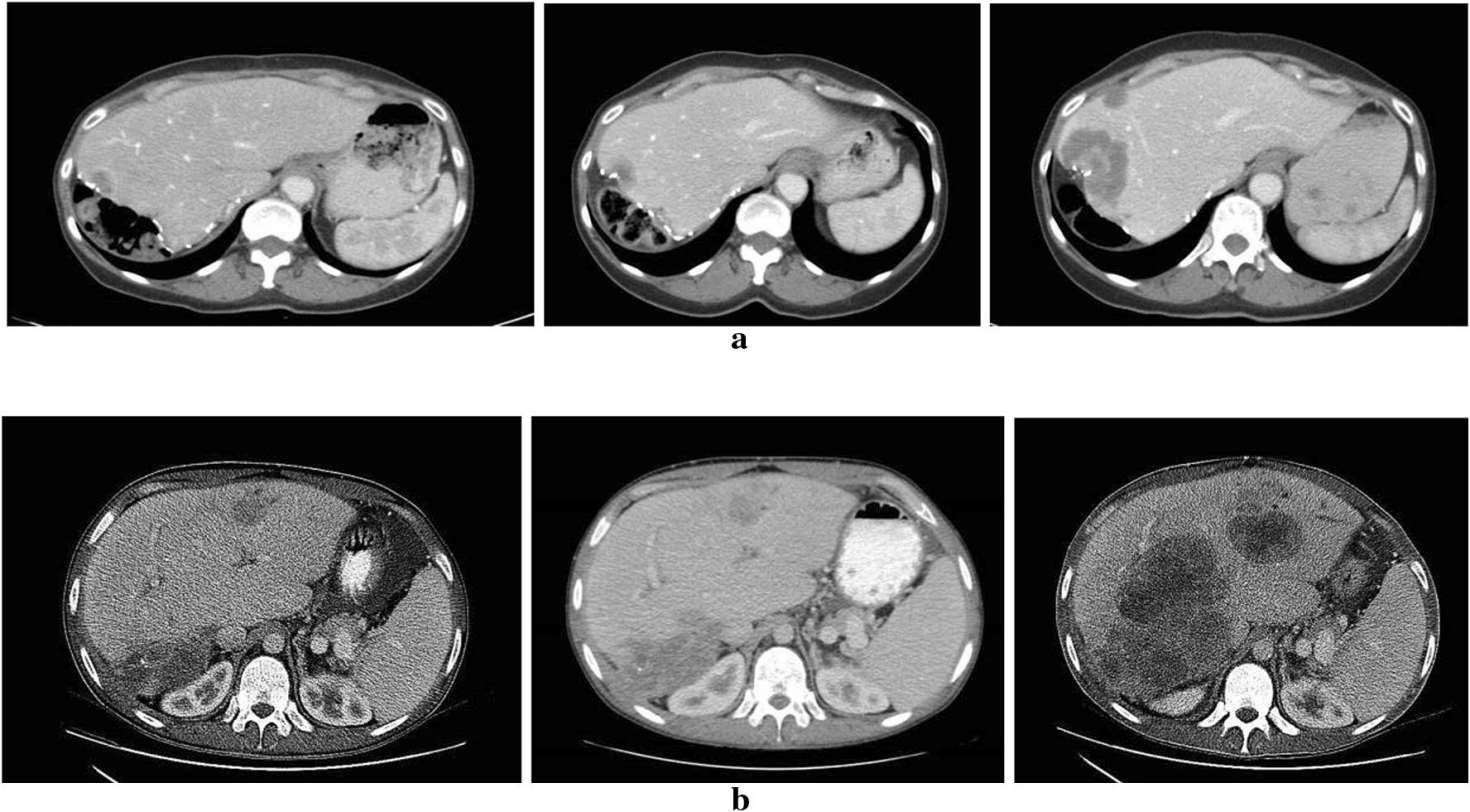
(**a**) CT-scan of abdomen in patient 102 before baseline (left panel), at baseline (middle panel) and first evaluation at week 8 (right panel). This patient fulfilled the criterion for hyperprogressive disease. (**b**) CT-scan of abdomen in patient 109 before baseline (left panel), at baseline (middle panel) and first evaluation at wee 8 (right panel). This patient fulfilled the criterion for hyperprogressive disease.

The phenomenon of cancer drug treatment related tumour hyperprogression (HP) has recently been observed and reported^19,20^. Since all patients radiologically evaluated in our study showed PD already at week 8 we calculated TGK (see above) while on Mbz treatment and compared it with the TGK on the treatment preceding the study treatment. These data are summarized in Fig. 1. Out of the 8 patients possible to analyze, 4 fulfilled the criteria for hyperprogressive disease suggested by Saâda-Bouzid^18^. Notably, as observed in the 3 patients with CT assessments following the stop of Mbz treatment and who had no further anti-cancer treatment, continued tumour progression at similar or higher speed as during Mbz was observed (Fig. 2). CT scans at pre-baseline, baseline, and while on/after Mbz treatment in 2 patients with colorectal cancer metastatic to the lung and liver and fulfilling criteria for hyperprogressive disease are shown in Fig. 3a,b.

#### Time to tumour progression, overall survival and performance status

**Figure 4:**
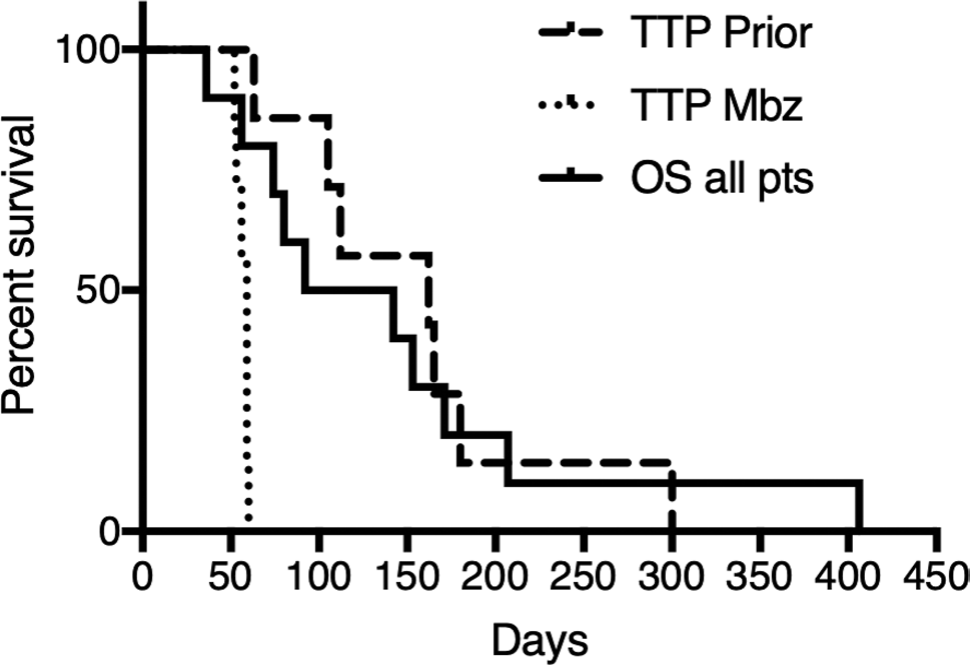
Time to tumour progression (TTP, days) while on the treatment preceding mebendazole (Mbz) and on treatment with Mbz for the patients (n = 8) included and evaluated by CT-scan at week 8. Overall survival (OS, days) are for all patients included and starting treatment phase (n = 10).

For radiologically evaluable patients TTP on the treatment preceding Mbz was median 162 and mean 155 (range 63–300) days while TTP on Mbz was median 59 and mean 57 (range 52–60) days. Overall survival (OS), defined as time in days from first dose of Mbz to death for the 10 patients starting treatment phase was median 117 and mean 142 (range 36–406) days. TTP and OS are shown in Fig. 4. None of the patients had longer TTP while on Mbz compared with that observed while on the treatment preceding Mbz.

At the pre-termination visit, no patient had WHO performance status grade 0 and only 1 patient had grade 1. Six patients had WHO performance status grade 2 and 2 patients grade 3. One patient died before the pre-termination visit was performed. Thus, there was a gradual decline in the patients’ performance status between screening and the pre-termination visit.

#### Safety

Overall Mbz was safe and well tolerated even at doses up to 4 g/day. All 11 patients included reported AEs and the majority were grades 1–2 (27 of 63 AEs; 43%). The vast majority of all AEs (59 of 63 AEs; 93.7%) were unlikely related to study treatment. The most commonly reported AEs were abdominal pain (4 patients; 4 events), decreased appetite (4 patients; 4 events), nausea (3 patients; 3 events) and vomiting (3 patients, 3 events). Nine SAEs (serious adverse event), of which 3 were fatal, were reported by 5 patients. No SAEs were considered related to study treatment. Patient 111 with a history of tumour bleeding and thrombosis was on tranexamic acid and developed a thrombotic stroke following the day after Mbz test dose. Based on these circumstances and the peak concentration (31 ng/ml) the stroke was considered unlikely related to Mbz. No dose limiting toxicities were observed.

There were no significant changes over time in vital signs, physical examinations, ECG or laboratory assessments (haematology/biochemistry) that were related to study treatment.

With respect to the administered dose of Mbz, there were no clear differences in the safety profile between patients who received the maximum allowed dose (4 g/day) and patients who received a lower dose of Mbz.

#### Pharmacokinetics

**Figure 5:**
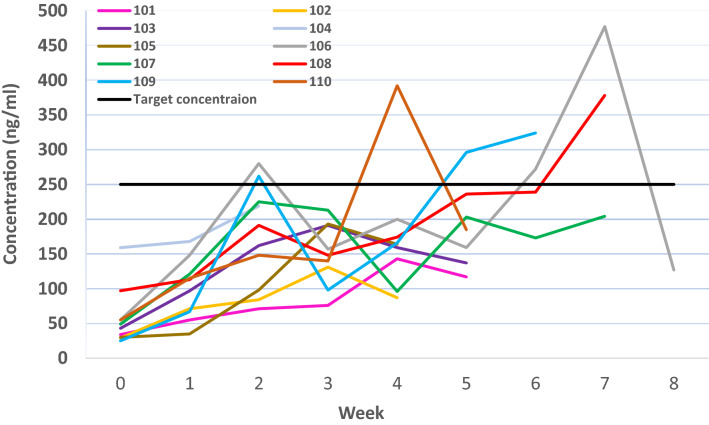
Graphical presentation of observed weekly serum-mebendazole concentrations during the pharmacokinetic (w 0) and treatment phases (w 1–8) for each of the study subjects. The horizontal line indicates the target concentration.

Despite administration of TDM based high doses of Mbz with the aim to reach and maintain a serum-Mbz concentration as close as possible to 300 ng/mL, only 4 patients occasionally reached this concentration during the treatment period. Two patients reached the target concentration for the first-time during study week 2, 1 patient during weeks 4 and 5, 2 patients during week 6 and 2 patients during week 7 (Fig. 5). The repeated measurements revealed that serum-Mbz concentrations were fluctuating despite continuous Mbz administration and only 2 patients reached the target concentration more than once during the treatment period. Mean maximum serum-Mbz concentration observed was 264 ng/mL (standard deviation 120, range 131–477). Five patients were prescribed the highest dose of Mbz allowed (4 g/day), yet only 2 of these patients reached the target serum-Mbz concentration. A detailed report on Mbz PK with PK modelling and assessment of Mbz metabolites will be reported separately.

#### Explorative analyses

Blood samples taken for exploratory research were analyzed for quantification of cytokines and other types of biomarkers. Peripheral blood mononuclear cells were collected to study the distribution of white blood cell types using flow cytometry. In particular, the aim was to investigate if Mbz could promote a shift from an M2 to an M1 phenotype of macrophages considered needed for triggering the immune system to promote an anti-tumoural effect. The results from the full exploratory analyses will be reported separately. Notably, the patient with clearly longest OS (406 days, patient 107) from start of Mbz, was the only one responding with increase in IFNg and IL-1b while on study. For the remaining patients, no obvious pattern of changes in cells or cytokines was observed.

## Discussion

All 10 patients who started continuous treatment with Mbz were withdrawn from study treatment between 14 and 91 days after the first dose due to rapidly PD and/or clinical deterioration. In light also of the difficulties to reach the target serum-Mbz concentration despite intensive TDM based dose adjustments allowing high daily Mbz doses, the study was stopped prematurely. Importantly, however, no safety issues were observed to influence this decision. Rather, it is concluded that Mbz is safe and tolerable at doses up to 4 g/day in patients with heavily pre-treated gastrointestinal cancer*.*

All patients showed rapid disease progression and 4 of the 8 patients with CT-evaluations even fulfilled criteria suggested for hyperprogressive disease. At this stage the relationship between exposure to Mbz and tumour growth stimulation resulting in HP is inconclusive. Mbz has not been shown to stimulate tumour cell growth in any experimental tumour model^21^. In this study, all patients were in progress when they were enrolled and most had poor prognostic features, e.g. disease resistant to several lines of chemotherapy, rapidly PD, high tumour burden, at study entrance, all making the possibility for tumour response very low and risk for rapid progression and short survival high.

However, since HP is seemingly related to treatment with immunologically active cancer drugs, but also to chemotherapy, in up to 25% of patients, it cannot be excluded that exposure to Mbz under specific circumstances might not only show lack of effect but might even result in HP^20^. Analysis of TGK after discontinuation of Mbz showed a ratio compatible with continuous HP. This could be explained by long lasting changes in tumour growth induced by Mbz or alternatively, it could mirror accelerated tumour growth as a part of natural course in the PD phase that can be observed in advanced cancer.

Speculatively, it might be that Mbz should be combined with a cytotoxic drug that stops tumour growth while an immune effect from Mbz allows time to act^22^. Thus, pending further analyses and investigations and despite the very promising prior preclinical and pilot clinical observations pointing to Mbz being highly prioritized for repositioning as a cancer drug^21^, given these results Mbz cannot be recommended for use in cancer outside of carefully designed clinical trials with close tumour growth surveillance to allow for early stop of treatment.

Despite TDM based individual dose escalation to the highest allowed dose Mbz, i.e. 4 g/day, our target serum-Mbz concentration considered therapeutic was achieved in only 4 patients and only temporarily and this was despite our effort to increase Mbz uptake by its administration together with fat from a standardized nutritional drink. Furthermore, the serum-Mbz concentrations showed great inter- and intraindividual variability. The reasons for these observations are unknown and various mechanisms could be considered, such as poor solubility, saturated absorption or induction of metabolism. Further analyses of PK data including Mbz metabolites might add mechanistic knowledge to this issue.

The problem to reach a high and stable drug exposure reasonably contributed considerably to the poor outcome in the present trial and overall, we conclude that Mbz is a promising pharmacophore but not the drug to bring forward in ongoing or future repositioning efforts. We rather suggest development of Mbz prodrugs with improved PK properties resulting in higher and less variable Mbz exposure. We have pilot in vivo data in mice indicating that such prodrugs are feasible to synthesize and development along this line has also been described recently^23^.

This study has strengths and limitations. Its key strength was the careful and intensive dosing and serum-Mbz adjustments and assessments making it possible for the first time to in more detail detect the Mbz PK shortcomings making it unsuitable for its repositioning into a cancer drug. Furthermore, the study suggests that Mbz is safe and, thus, suitable to bring forward as a promising pharmacophore for cancer treatment once detailing of PK/PD issues have been elucidated. Key limitations are the small number of patients included and with different tumour types making generalizability of results low and the basis for explorative analyses to expand the mechanistic and therapeutic knowledge on pros and cons of Mbz as a cancer drug poor. Furthermore, since all patients included in this study had advanced cancer progressive after standard treatment, the time on experimental treatment was short, which also adds to the difficulties to make firm conclusions of Mbz as a cancer drug.

In conclusion, single drug individualized dosed Mbz had no anti-cancer effect in therapy refractory gastrointestinal cancer. Still, based on the strong preclinical data, some very early clinical observations and its great tolerability, Mbz remains an interesting candidate for repositioning into a cancer drug. However, the molecule needs to be refined to improve on its PK properties and it might be that its potential anti-tumour properties will only emerge in patients with less advanced disease and/or in other cancer diagnoses known to be more responsive to immunotherapy. Alternatively, in combination with other cancer drugs, e.g. checkpoint blockade antibodies that release the effect of T cells, or other immunotherapeutics that promote cellular immunity and may benefit from a reduced M2 macrophage phenotype.

## Supplementary Information

Supplementary Information.

## Data Availability

The data collected and analysed during this study are available from the corresponding author on reasonable request.

## References

[1] Witassek F, Burkhardt B, Eckert J, Bricher J (1981). Chemotherapy of alveolar echinococcosis. Eur. J. Clin. Pharmacol..

[2] Woodtli W (1985). Effect of plasma mebendazole concentrations in the treatment of human echinococcosis. Am. J. Trop. Med. Hyg..

[3] Luder PJ, Siffert B, Witassek F, Meister F, Bricher J (1986). Treatment of hydatid disease with high oral doses of mebendazol. Eur. J. Clin. Pharmacol..

[4] Mukhopadhyay T, Sasaki J, Ramesh R, Roth JA (2002). Mebendazole elicits a potent antitumor effect on human cancer cell lines both in vitro and in vivo. Clin. Cancer. Res..

[5] Nygren P, Fryknäs M, Agerup B, Larsson R (2013). Repositioning of the antihelmintic drug mebendazole for the treatment of colon cancer. J. Cancer. Res. Clin. Oncol..

[6] Bai R-Y, Staedtke V, Aphrys CM, Gallia GL, Riggins GJ (2011). Antiparasitic mebendazole shows survival benefit in 2 preclinical models of glioblastoma multiforme. Neuro Oncol..

[7] Doudican NA, Byron SA, Pollock PM, Orlow SJ (2013). XIAP downregulation accompanies mebendazole growth inhibition in melanoma xenografts. Anticancer Drugs.

[8] Dobrosotskaya I, Hammer G, Schteingart D, Maturen K, Worden F (2011). Mebendazole montherapy and long-term disease control in metastatic adrenocortical carcinoma. Endocr. Pract..

[9] Nygren P, Larsson R (2014). Drug repositioning from bench to bedside: Tumour remission by the antihelmintic drug mebendazole in refractory metastatic colon cancer. Acta Oncol..

[10] Sasaki J (2002). The antihelminitic drug mebendazole induces mitototic arrest and apoptosis by depolymerizing tubulin in small-cell lung cance cells. Mol. Cancer Ther..

[11] Tan Z, Chen L, Zhang S (2016). Comprehensive modeling and discovery of mebendazole as a novel TRAF2- and NCK-interacting kinase inhibitor. Sci. Rep..

[12] Larsen AR (2015). Repurposing the antihelmintic mebendazole as a hedgehog inhibitor. Mol. Cancer Ther..

[13] Blom K (2017). The anticancer effect of mebendazole by be due to M1 monocyte/macrophage activation via ERK 1/2 and TLR8-dependent inflammasome activation. Immunopharmacol. Immunotoxicol..

[14] Blom K (2019). Mebendazole-induced M1 polarisation of THP-1 macrophages may involve DYRK1B inhibition. BMC Res. Notes.

[15] Rubin J (2018). Mebendazole stimulates CD14+ myeloid cells to enhance T-cell activation and tumour cell killing. Oncotarget.

[16] Braithwaite PA, Roberts MS, Allan RJ, Watson TR (1982). Clinical pharmacokinetics of high dose mebendazole in patients treated for cystic hydatid disease. Eur. J. Clin. Pharmacol..

[17] WHO (1996). Guidelines for treatment of cystic and alveolar echinococcosis in humans. Bull. World Health Org..

[18] Saâda-Bouzid E (2017). Hyperprogression during anti-PD-1/PD-L1 therapy in patients with recurrent and/or metastatic head and neck squamous cell carcinoma. Ann. Oncol..

[19] Champiat S (2018). Hyperprogressive disease: Recognizing a novel pattern to improve patient management. Nat. Rev. Clin. Oncol..

[20] Ferrara R (2018). Hyperprogressive disease in patients with advancec non-small cell lung cancer treated with PD-1/PD-L1 inhibitors or with single-agent chemotherapy. JAMA Oncol..

[21] Guerini AE (2019). Mebendazole as a candidate for drug repurposing in oncology: An extensive review of current literature. Cancers.

[22] Rushworth LK (2019). Repurposing screen identifies mebendazole as a clinical candidate to synergize with docetaxel for prostate cancer treatment. Br. J. Cancer.

[23] Zimmermann SC (2018). N-substituted prodrugs of mebendazole provide improved aqueous solubility and oral bioavailability in mice and dogs. J. Med. Chem..

