# Supplementary material for: A phase 2a clinical study on the safety and efficacy of individualized dosed mebendazole in patients with advanced gastrointestinal cancer
Source: Sci Rep. 2021 Apr 26;11:8981. doi: 10.1038/s41598-021-88433-y (PMC8076239; doi:10.1038/s41598-021-88433-y)
Supplement: Supplementary file 1 — Supplementary Information. [file 41598_2021_88433_MOESM1_ESM.pdf]

A phase 2a clinical study on the safety and efficacy of individualized dosed mebendazole in patients with advanced gastrointestinal cancer

S Mansoori[1*], M Fryknäs[1], C Alvfors[2], A Loskog[3,4], R Larsson[1] and P Nygren[3]

Supplementary tables

Supplementary table 1. Algorithm for the starting dose of mebendazole (Mbz) in the treatment phase (based on PK after a single 100 mg Mbz test dose).

**Mbz C_{max} following single PK dose Mbz 100 mg**	**Repeat dose Mbz to be started**
>400 ng/mL	None, patient off study
201 - 400 ng/mL	50 mg x 1
101 - 200 ng/mL	50 mg x 2
68 - 100 ng/mL	100 mg x 2
51 - 67 ng/mL	150 mg x 2
34 - 50 ng/mL	200 mg x 2
26 - 33 ng/mL	300 mg x 2
21 - 25 ng/mL	400 mg x 2
11 - 20 ng/mL	500 mg x 2
≤ 10 ng/mL	1000 mg x 2

Supplementary table 2. Algorithm for dosing of mebendazole (Mbz) during treatment phase (based on PK results after repeated dosing)

**Steady state C_{max} for Mbz**	**Mbz dose adjustment based on the preceding PK results**
≤50 ng/mL	6x[1]
51 - 100 ng/mL	3x
101 - 200 ng/mL	1.5x
201 - 249 ng/mL	1.25x
250 - 350 ng/mL	No change
351 - 400 ng/mL	0.75x
401 - 600 ng/mL	0.5x
601 - 1200 ng/mL	Stop until ≤ 350 ng/mL, then 0.25x
> 1200 ng/mL	Stop. Patient goes off study

[1] x denotes current dose that resulted in the left column C_{max}. In the case the dosing algorithm suggested a daily dose not possible to divide in two equal doses, this was accepted and the highest dose should be administered in the morning
